# Regoaling: a conceptual model of how parents of children with serious illness change medical care goals

**DOI:** 10.1186/1472-684X-13-9

**Published:** 2014-03-13

**Authors:** Douglas L Hill, Victoria Miller, Jennifer K Walter, Karen W Carroll, Wynne E Morrison, David A Munson, Tammy I Kang, Pamela S Hinds, Chris Feudtner

**Affiliations:** 1 The Children’s Hospital of Philadelphia, Philadelphia, PA, USA; 2 Children’s National Health Systems, Washington, DC, USA; 3 The Children’s Hospital of Philadelphia, General Pediatrics – 3535 Market Street, Room 1523, 34th, Civic Center Boulevard, Philadelphia, PA 19104, USA

**Keywords:** Parental decision making, Pediatric palliative care, Goals, Disengagement, Reengagement, Regoaling, Positive affect, Negative affect, Hopeful thinking, Conceptual model

## Abstract

**Background:**

Parents of seriously ill children participate in making difficult medical decisions for their child. In some cases, parents face situations where their initial goals, such as curing the condition, may have become exceedingly unlikely. While some parents continue to pursue these goals, others relinquish their initial goals and generate new goals such as maintaining the child’s quality of life. We call this process of transitioning from one set of goals to another regoaling.

**Discussion:**

Regoaling involves factors that either promote or inhibit the regoaling process, including disengagement from goals, reengagement in new goals, positive and negative affect, and hopeful thinking. We examine these factors in the context of parental decision making for a seriously ill child, presenting a dynamic conceptual model of regoaling. This model highlights four research questions that will be empirically tested in an ongoing longitudinal study of medical decision making among parents of children with serious illness. Additionally, we consider potential clinical implications of regoaling for the practice of pediatric palliative care.

**Summary:**

The psychosocial model of regoaling by parents of children with a serious illness predicts that parents who experience both positive and negative affect and hopeful patterns of thought will be more likely to relinquish one set of goals and pursue a new set of goals. A greater understanding of how parents undergo this transition may enable clinicians to better support them through this difficult process.

## Background

Parents of seriously ill children confront an extremely difficult, and at times emotionally overwhelming, situation: their child may die in the near future or live with a severely debilitating chronic condition, become medically fragile, and often require intensive medical treatment and technology to remain stable. In the midst of these daunting circumstances, parents (as the primary decision makers for the child, and potentially including step-parents or other guardians) must join with clinicians to make important and complex decisions about their child’s medical care. These decisions are based on a variety of considerations – including medical information [1,2], values [3], cultural expectations [4], and parental beliefs [5] – and are influenced by various factors – including parental emotional states and patterns of thinking [6-8]. All of these considerations and influences combine to shape the hopes that parents of seriously ill children have for the child and the goals of medical care.

Parent hopes and goals may change over time as the child’s condition changes [9]. Some parents of children with serious illness are confronted with evidence that their initial goals (such as curing the condition and restoring their child to full health) are no longer realistic. At that point, these parents face a decision point with two potential paths. On the first path, parents may persist in the pursuit of their initial goals even though they are aware that the attainment of these goals is believed by members of the clinical team to no longer be realistic. On the second path, parents may relinquish or disengage from their initial goals for the child’s medical care, and pursue or reengage in a set of new goals viewed now as more achievable, appropriate, or desirable by the parents, such as managing the child’s condition with the least amount of treatment-related pain or suffering, limiting exposure to invasive or extreme interventions, and maintaining the child’s quality of life. Note that the new goals adopted by parents may or may not correspond with the goals of the medical team.

We call the process in the second path “regoaling” (see Table 1 for brief definitions of key concepts). We do not believe that all parents in this situation *can* or *should* go through this regoaling process. Rather, our intention is to examine this second path of regoaling, and to review factors that may inhibit or facilitate the regoaling process for parents of seriously ill children.

**Table 1:** Brief definitions of key concepts

**Concept**	**Definition**
**Serious illness**	Life threatening medical conditions that are highly likely to cause death in the near future, or medical conditions likely to result in severe disability, medical fragility, or dependency upon medical treatments and technology for survival.
**Children**	Persons less than 18 years of age, and for the purposes of this paper, also cognitively impaired patients older than 18 years who are seriously ill and unable to make medical decisions for themselves.
**Parents**	Adults who are the primary medical decision makers for the child, including step-parents, foster parents, grandparents, and other family members.
**Goals**	Aspirational expectations or hopes for the child’s care or future.
**Goal disengagement**	Accepting that a goal (or set of goals) is no longer desirable, appropriate, or achievable, and discontinuing efforts to achieve the goal (or set of goals).
**Goal reengagement**	Adopting a new goal (or set of goals) and initiating efforts to achieve the new goal (or set of goals).
**Regoaling**	The process over time of disengaging from one set of goals and reengaging or adopting a new goal (or set of goals).
**Positive affect**	The experience of a general positive mood or specific positive emotions.
**Negative affect**	The experience of a general negative mood or specific negative emotions.
**Hopeful thinking**	A sense of being generally successful in achieving personal goals (personal agency) and being able to generate strategies to achieve these goals (pathways).

Research studies have not yet focused on the regoaling process, neither describing how goals change over time nor the factors that influence the regoaling process. Understanding this regoaling process is important for at least three reasons. First, if parent goals are changing over time, health care providers need to recognize the new goals. Second, a better understanding of the regoaling process may allow health care providers to facilitate the process when appropriate. Third, being familiar with the process of regoaling may help providers manage disagreements about goals by giving parents time to work their way through the regoaling process rather than directly challenging their initial goals.

In this article, we first identify factors relevant to the regoaling process including disengagement from goals, reengagement in new goals, positive and negative affect, and hopeful thinking and apply these factors to the context of parental decision making for a seriously ill child. Second, we integrate these factors into a dynamic conceptual model of regoaling by parents of children with serious illness. Third, we present four research questions that will be empirically tested in an ongoing longitudinal study of decision making among these parents. Finally, we describe the clinical implications of the regoaling conceptual model to the practice of pediatric palliative care.

## Discussion

### Goal disengagement

Commitment to valued personal goals or strivings that are meaningful and potentially attainable is associated with subjective well-being and life satisfaction [10,11]. Goals are also an important part of life schemes, cognitive representations of a person’s life that provide a sense of order and purpose [12]. Individuals are motivated to meet personal goals, and perceived failure to make progress toward important goals can cause negative affect such as feelings of anxiety, dysphoria, or despair [11,13,14]. While negative affect may initially lead to increased efforts to meet a goal, if a goal is consistently or permanently blocked, the negative affect may become overwhelming [12,15]. In this situation, some individuals may disengage from their initial goal, accepting that the goal is no longer desirable, appropriate, or achievable, and discontinue their efforts to achieve the goal. Some studies have found that the ability to disengage from unrealistic goals may be associated with lower levels of depression, higher levels of subjective well-being, and better physical health [16-18].

There are few goals as highly valued and central to a parent’s self-concept as wanting a child to be healthy and happy [19]. When parents learn that their child has an incurable condition, some will start to focus on new goals such as managing their child’s condition or minimizing their child’s suffering. Other parents, however, may not set aside their initial goals and continue to request aggressive treatments that place the child at risk for increased suffering with little or no chance of medical benefit [7].

The few goal disengagement studies looking at parents of children with serious illness (children with cancer, family members with mental illness), found that those who reported being able to disengage from previous goals and engage in new ones had better long term outcomes [17,20]. While these studies did not document what specific goals the parents disengaged from or engaged in, the authors did suggest that the parents with better outcomes focused on self-defined high-priority goals of taking care of the sick child or family member while relinquishing lower priority goals (such as the parent’s career goals). To our knowledge no studies have examined factors that promote or hinder parents of seriously ill children from disengaging from the initial set of high priority goals directly related to their child’s health when these goals are no longer realistic.

Based on theories of goal regulation and our clinical experience, we suggest that disengagement may be facilitated by at least three factors. First, if increased goal-directed efforts are unsuccessful, negative affect may serve as an alarm signal that previously established goals need to be reevaluated [15,21] and disengaged [14,17,22]. Second, disengagement is more likely if an individual has a high degree of certainty that the goal is unattainable and that persistence in attempting to achieve the goal will have negative consequences [16]. Third, the presence of feasible alternatives to the initial goal increases the likelihood of disengagement from the unattainable goal [23,24].

Disengagement from goals that are no longer realistic or helpful may be hindered by at least four factors. First, the majority of individuals have moderate positive illusions [25,26], objectively unrealistic beliefs that positive things such as being healthy are more likely to happen to them and that negative events such as becoming ill will not happen personally to them or their child [27]. In many contexts, milder illusions, while factually inaccurate, appear to be adaptive in that they encourage individuals to persist at difficult goals and help individuals cope with negative situations such as breast cancer [28]. While very common, beyond a certain point such illusions may lead to maladaptive persistence in unattainable goals [26,29]. In the context of children with serious illness, parents often believe either that a child with a poor prognosis will recover or that, regardless of likelihood of recovery, parents have a duty to stay positive and pursue any possibility of a cure [30,31].

A second factor that may inhibit goal disengagement is perceived sunk costs [32,33]. Individuals who have invested time, money, and effort into medical treatment may be reluctant to change that decision [34]. Parents of seriously ill children may continue with aggressive treatments because giving up would be admitting that the child and family have suffered seemingly for nothing.

A third factor that may hinder goal disengagement is the depletion of self-regulation resources - the psychological ability to examine and exert effort to change pre-existing patterns of thought, emotion, and behavior [35]. Individuals sometimes make poor decisions if they do not have sufficient time to replenish self-regulation resources [36,37]. Parents of seriously ill children often report feeling physically and emotionally depleted [38] and may not have sufficient self-regulation resources to reevaluate their initial goals.

A fourth factor that may interfere with goal disengagement is the degree to which caring for the child is central to the parent’s self-concept. Some parents of children with serious illness have relinquished almost everything else to focus on caring for their sick child. These parents may have difficulty disengaging from goals such as curing their child because they are unable to picture themselves doing anything other than caring for the child.

### Goal reengagement

We define goal reengagement as adopting a new goal (or set of goals) and initiating efforts to achieve the new goal (or set of goals). Theories of goal regulation suggest that engaging in new goals can reduce the distress associated with giving up on highly valued, unattainable goals and offset the negative consequences of continuing to persist in an unattainable goal [17]. The ability to disengage from previous goals and the ability to reengage in new goals are somewhat independent [17]. The evidence for the benefits of goal reengagement is mixed, with some studies finding associations between reengagement and lower levels of depression and higher levels of purpose in life [17,20], and others finding that reengagement predicts well-being only for individuals who have trouble disengaging from goals [17,18]. In some cases reengagement was actually associated with higher levels of distress [20].

Research on goal regulation has not yet clarified what facilitates or inhibits goal reengagement. Some researchers have suggested that personality factors such as optimism and perceived control may predict the ability to engage in new goals [22]. Plausibly, some of the factors that facilitate goal disengagement (availability of alternatives, certainty that the initial goals are unattainable) would also predict goal reengagement. Individuals who go through a negative, traumatic experience, such as having a baby with a serious illness or bereavement, often generate new beliefs, perspectives, and goals to cope with the experience [39-41]. What helps some individuals generate new goals in these circumstances is, however, unclear.

### Affect and regoaling

Affect is a broad term that refers to a general mood or a specific emotional experience [42]. The current conceptualization of affect views positive affect (e.g., a general good mood or a specific positive emotion such as joy) and negative affect (e.g., a general negative mood or a specific negative emotion such as anxiety or anger) as separate emotional experiences rather than opposite ends of the same continuum [43-45]. This is consistent with studies finding that even in extreme situations, caregivers experience both positive and negative affect [6,46,47]. In addition to feelings of sadness and distress about their child’s condition, parents of seriously ill children report being overwhelmed with feelings of love for the child and of gratitude for the support they have received.

Negative affect is associated with more systematic, in-depth processing of available evidence and rumination [42]. As noted above, some level of negative affect and in-depth processing may facilitate reevaluation of unattainable goals and disengagement [22]. Too much negative affect, however, may lead to despair and complete withdrawal from the situation [15].

Positive affect, in contrast, usually serves as a sign that things are going well [15] and thus may hinder regoaling, but individuals in a positive mood are also capable of paying attention to negative information that is important and self-relevant [48,49]. Positive affect may play an important role in the regoaling process in at least three ways. First, positive affect may increase the accessibility of memories of other positive experiences [50] and facilitate the ability to think creatively and flexibly [51,52]. Individuals experiencing positive affect may be able to “broaden and build,” stepping back to see the larger picture and come up with new ideas and strategies [53]. Second, positive affect may help replenish depleted self-regulatory resources [36]. Third, positive affect is associated with approach goals, which are goals that individuals work toward in order to gain or accomplish something positive, as opposed to goals that seek to avoid a negative outcome [54].

Some studies suggest that individuals who experience both positive and negative emotions while coping with a severe stressor experience better psychological outcomes [46,55-57]. These findings suggest that a balance of both positive and negative affect may help individuals cope with negative events and transition from one set of goals to another.

### Hopeful thinking

Another factor, closely tied to affect, that may influence regoaling is hopeful thinking. Hope has long been recognized as important when individuals and families are coping with a serious illness [58]. Moreover, being hopeful is not the same thing as being unrealistic or in denial. Parents of seriously ill children report continuing to be hopeful while understanding their child’s dire medical prognosis [9].

While there are many different definitions of hope in the health care setting [59], our research team uses the definition of hope developed by C.R. Snyder, in which hope is a set of goal-directed cognitive processes that influence and are influenced by emotion. The theory has two major parts: “Agency” is an individual’s sense of being generally successful in meeting goals. “Pathways” is an individual’s sense of being able to generate successful plans to achieve those goals [60,61]. High hope individuals have high levels of both agency and pathways, tend to generate more goals overall, are better at working to achieve their goals, are more likely to think of new ways of achieving a blocked goal, and are more likely to substitute another goal for a blocked goal [61-64].

High hope individuals experience less negative and more positive emotion when they are unable to achieve a goal. In contrast, low hope individuals are more likely to experience negative emotions after a setback, are more likely to give up, and are less able to set new goals. In other words, individuals high in hopeful thinking may be better at regoaling than individuals who are low in hopeful thinking. Hope theory has been supported by studies finding that high hope individuals have better psychosocial outcomes after burn injuries [65], report higher levels of well-being after the death of a loved one [66], experience better adjustment after spinal cord injuries [67], and are less likely to experience distress when taking care of children with chronic health conditions [68].

We examined hopeful thinking in a study of parents of children receiving palliative care and found that these parents had levels of hope comparable to other populations (i.e., patterns of hopeful thinking do not disappear even in the face of this grim situation) [6]. We also found that as the child’s perceived health status worsened, parents with higher levels of hope were more likely to decide to limit interventions, such as by having a do-not-attempt-resuscitation order (DNAR) [6]. These findings suggest that hopeful thinking is not a form of denial for these parents, and high hope parents may be better at transitioning from one set of goals (help my child recover) to another (keep my child comfortable).

### Conceptual model

**Figure 1:**
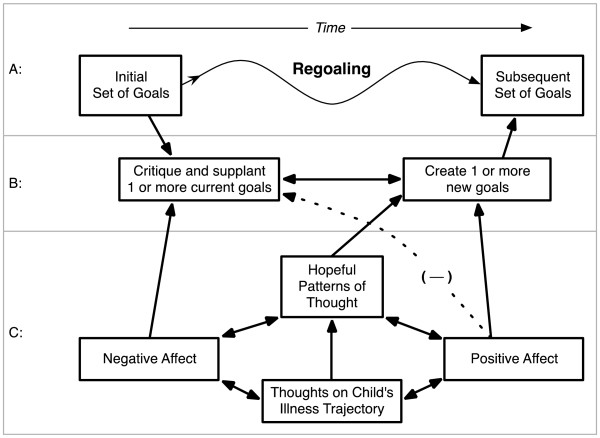
Regoaling process and underlying influential factors.

Based on our previous research and clinical experience, we have developed a dynamic conceptual model of regoaling by parents of children with serious illness. The primary question this model attempts to address is this: As a child’s condition worsens and the chances of full recovery become less likely, how do some of these parents make the transition from one set of goals (e.g., find a treatment that will cure my child) to another (e.g., make my child’s final days meaningful). Central to this model is the idea that parent treatment goals and priorities for sick children may change over time (Figure 1, top panel A), and regoaling is a beneficial event for many (but not all) of these parents.

For the sake of simplicity this model portrays regoaling as a one-way transition from an initial set of goals to a subsequent set of goals. The actual process often is not linear but circuitous, looping back and forth between initial goals and new goals, developing new goals while still maintaining initial goals (such as to keep fighting for a cure while also focusing on keeping the child comfortable), or transitioning sequentially over time through multiple sets of new goals.

The proposed model suggests there are two processes underlying regoaling (Figure 1, middle panel B): 1) critiquing and letting go of the initial goals (disengagement) and 2) generating new goals (reengagement). The model then identifies several factors that influence the critiquing of initial goals and the generation of new goals, including: 1) negative affect, 2) positive affect, and 3) hopeful thinking (Figure 1, bottom panel C).

Our model begins with thoughts about the child’s current illness trajectory (Figure 1, panel C) (e.g., is the child getting better or worse?). If the child is getting better, the parent will be more likely to experience positive mood and less likely to question the initial set of goals. If the child’s condition is getting worse, the parent will experience negative affect and be more likely to question their initial assumptions. The parents will increase their efforts to meet the initial goals and look for other treatments or approaches that will help the child. If these efforts fail, over time some of these parents will be more likely to accept that their initial goals are unrealistic and start to disengage from them.

Some level of positive affect and hopeful thinking may help parents of seriously ill children manage the negative affect associated with giving up on a valued goal and help them start to think about new goals. A testable aspect of our model is that both negative and positive affect are necessary for regoaling. Parents who experience only negative affect are more likely to be overwhelmed with despair and relinquish their initial goals related to their child’s care without generating any new goals. Parents who experience only positive affect are unlikely to give up on their initial goals or consider new goals (hence the negative dashed line between positive affect and critiquing initial goals).

In our model, we suggest that parents of children with serious illness with higher levels of hopeful thinking are more likely to disengage from initial goals, experience more positive and less negative affect, and generate new goals. Over time, this will be a reinforcing cycle. Experiencing some level of positive affect will increase hopeful thinking, hopeful thinking will increase the likelihood of generating new goals, and focusing on new goals will help reduce negative affect and increase positive affect, which in turn will increase hopeful thinking. This cycle leads to the transition from the initial set of goals to a new set of goals.

### Future research directions

This conceptual model has led us to examine four research questions that we are investigating in our ongoing prospective longitudinal study of decision making among parents of children with life-threatening illness. We have enrolled 200 parents and are collecting data about their goals seven times over the course of two years. This study will address questions related to regoaling including:

1) Does regoaling occur for parents of children with serious illness? Some retrospective evidence of regoaling exists, as parents of children who have died and parents of children with cancer have reported that their goals have changed over time [31,69]. These data, though, are vulnerable to recall biases and constructed memories. Only one study to our knowledge has recorded parent goals or hopes at different time points to see whether the goals have indeed changed as the child’s condition changes [9].

2) What does the process of regoaling look like over time for parents of children with serious illness? Do these parents rapidly switch from one set of goals to another after receiving new information about their child’s condition, or is there a more gradual process of shifting from one set of goals to another?

3) What motivates regoaling by these parents? Do differences in psychological factors among parents like affect and hope increase the likelihood of regoaling?

4) Do parents of seriously ill children who go through the regoaling process experience better outcomes (e.g., less anxiety and depression) than those who maintain their initial goals?

### Potential clinical implications

This model has three potential implications for healthcare providers caring for children with serious illness. First, providers must be aware that not all parents can or should go through this regoaling process. Some parents will persist in their initial goals regardless of what events or conversations unfold, and for some this may be the best way to maintain engagement in their child’s care and hope for the future. In other cases, parents of children with serious illness may cling to their initial goals because they see no alternatives, and the thought of relinquishing their initial goals for their child creates high levels of anxiety, depression, and despair.

Second, providers may be able set the stage for regoaling by providing clear information about the child’s condition, giving parents time to accept the news, and assessing their understanding. Providers can acknowledge and support parents’ emotional reactions (“I can see how hard this is”), and can create a small positive experience by praising them and recognizing how much they are doing (“I can see how much you and your husband care about your son”) [70]. Providers may be able to facilitate other periods of positive affect by showing that they care, encouraging parents to seek social support, reminding them to take care of themselves as well as the child, and making sure they have an opportunity to freely express what they are going through without judgment.

Third, parents of seriously ill children who show a mix of positive and negative emotions may be closer to being ready to discuss new goals. Those parents who show only positive affect, only negative affect, or no affect at all may need additional time and support before discussing new goals. When parents seem ready, healthcare providers can gently prompt them to think about new hopes and goals by asking what they are hoping for given the current situation. Some parents may immediately identify other goals, such as taking the child home, reducing the number of painful interventions, or having a baptism. Others may need time to think of new goals. Providers may be able to provide suggestions (“What some loving families have done in this situation is . . .”). Once parents start to talk about new goals, providers can focus on the most realistic possibilities and offer suggestions of how those goals can be achieved. They can help these parents achieve hopeful thinking by supporting both pathways (“here are ways you can achieve this goal, and here is how we can help”) and agency (“I know this is hard, but you will be able to do this”).

## Summary

The psychosocial model of regoaling by parents of children with a serious illness predicts that parents who experience both positive and negative affect and hopeful patterns of thought will be more likely to relinquish one set of goals and pursue a new set of goals. A greater understanding of what helps these parents achieve this transition may enable clinicians to better support parents though this difficult process.

## Competing interests

The authors declare that they have no competing interests.

## Authors’ contributions

All authors made substantive intellectual contributions to conception and design of this conceptual model and manuscript. DH and CF were the lead authors responsible for initial drafting of the manuscript. VM and JW revised the manuscript critically for important intellectual content. All authors read and approved the final manuscript.

## Pre-publication history

The pre-publication history for this paper can be accessed here:

http://www.biomedcentral.com/1472-684X/13/9/prepub
